# Passing through – reasons why migrant doctors in Ireland plan to stay, return home or migrate onwards to new destination countries

**DOI:** 10.1186/s12960-016-0121-z

**Published:** 2016-06-30

**Authors:** Ruairí Brugha, Sara McAleese, Pat Dicker, Ella Tyrrell, Steve Thomas, Charles Normand, Niamh Humphries

**Affiliations:** Department of Epidemiology and Public Health Medicine, Royal College of Surgeons, Dublin, Ireland; Centre for Health Policy and Management, Trinity College Dublin, Dublin, Ireland

**Keywords:** Career progression, Foreign-trained doctors, Health workforce, Onward migration, WHO Global Code

## Abstract

**Background:**

International recruitment is a common strategy used by high-income countries to meet their medical workforce needs. Ireland, despite training sufficient doctors to meet its internal demand, continues to be heavily dependent on foreign-trained doctors, many of whom may migrate onwards to new destination countries. A cross-sectional study was conducted to measure and analyse the factors associated with the migratory intentions of foreign doctors in Ireland.

**Methods:**

A total of 366 non-European nationals registered as medical doctors in Ireland completed an online survey assessing their reasons for migrating to Ireland, their experiences whilst working and living in Ireland, and their future plans. Factors associated with future plans – whether to remain in Ireland, return home or migrate to a new destination country – were tested by bivariate and multivariate analyses, including discriminant analysis.

**Results:**

Of the 345 foreign doctors who responded to the question regarding their future plans, 16 % of whom were Irish-trained, 30 % planned to remain in Ireland, 23 % planned to return home and 47 % to migrate onwards. Country of origin, personal and professional reasons for migrating, experiences of training and supervision, opportunities for career progression, type of employment contract, citizenship status, and satisfaction with life in Ireland were all factors statistically significantly associated with the three migratory outcomes.

**Conclusion:**

Reported plans may not result in enacted emigration. However, the findings support a growing body of evidence highlighting dissatisfaction with current career opportunities, contributing to the emigration of Irish doctors and onward migration of foreign doctors. Implementation of the WHO Global Code, which requires member states to train and retain their own health workforce, could also help reduce onward migration of foreign doctors to new destination countries. Ireland has initiated the provision of tailored postgraduate training to doctors from Pakistan, enabling these doctors to return home with improved skills of benefit to the source country.

**Electronic supplementary material:**

The online version of this article (doi:10.1186/s12960-016-0121-z) contains supplementary material, which is available to authorized users.

## Background

The effectiveness of the international recruitment of doctors as a sustainable strategy to meet the needs of destination countries has been questioned [1]. Evidence casts doubt on the assumption that the choice available to migrating doctors lies between settling in the first destination country or returning ‘home’ after a period of practice or training [2]. Doctor migration has been described, instead, as a ‘carousel’ [3], where doctors move from one destination country to the next in search of further advantages, or in response to disappointments in their initial destination country. However, there has been little empirical research on the likelihood of onward migration by doctors and the factors that might determine this. Many high-income English-speaking countries, notably the United States, United Kingdom, Australia and Canada [4], rely heavily on international recruitment to meet their health workforce needs. Ireland is an example of such a destination country, although it has received less attention until recently [5, 6]. In Ireland, however, onward migration by foreign-trained doctors to larger English-speaking countries appears to be occurring [7].

Between 2000 and 2007, the proportion of foreign-trained doctors registered with the Medical Council of Ireland increased from 13 % to 32 % [5], plateauing at between 30 % and 35 % between 2007 and 2012 [8]. This was the result of a 2- to 3-fold increase between 2000 and 2010 in doctors migrating to Ireland from countries such as Pakistan and India, and larger rates of increase from African countries such as Sudan, Nigeria and South Africa [9]. Emergency active recruitment campaigns by Ireland’s National Health Service Executive were used to recruit doctors from Pakistan in 2011 and 2013, attracting local media attention [10]. In 2007, Ireland doubled the annual intake of Irish/European Economic Area (EEA) nationals into its medical schools from a base of 305 to the 725 needed for medical workforce self-sufficiency [11, 12]. The target intake was reached by 2011, mainly through a new 4-year Graduate Entry Medicine Programme [13], and approximately 720 EEA (almost all Irish) nationals graduated in 2015, along with approximately 400 non-EEA nationals. Irish medical schools have a long tradition of training high fee-paying non-EEA nationals, most of whom are assumed to return to their countries of origin. Ongoing international recruitment, supplemented by the upward trend in local graduates since 2011, has masked the growing rate of emigration of Irish doctors; this trend has been previously discussed in a study providing a graphic insight into the scale and reasons for the emigration of Irish-trained health professionals [14].

Qualitative research involving foreign-trained doctors in Ireland revealed the widespread disappointment and frustration among doctors who had come to Ireland seeking further training and career advancement [7]. Many reported working in ‘service posts’ that did not offer formal postgraduate training or career progression opportunities, described by respondents as ‘dead-end posts’. These qualitative findings informed the quantitative follow-up study reported herein, measuring foreign doctors’ future intentions, namely whether to remain in Ireland, return home or migrate onwards. This study thereby tests Zubaran’s [1] hypothesis that foreign doctors are a potentially mobile and transient component of a destination country’s medical workforce. The present paper reports the association between the reasons for foreign doctors to migrate to Ireland and their experiences while working and living in Ireland, as well as their migration intentions.

The broader term ‘foreign doctor’ is used herein to indicate non-Irish/EEA nationals who trained outside of Ireland, often termed international medical graduates, as well as non-Irish/EEA nationals who had graduated from Irish medical schools. The Ethics Committee of the Centre of Health Policy and Management and Centre for Global Health Research at Trinity College Dublin granted ethics approval for the study.

## Methods

An online survey of foreign doctors working in Ireland was conducted in April-May 2013 using Survey Monkey. The questions were informed by a similar earlier study on migrant nurses [15] and the results of the earlier qualitative phase [7]. The survey was piloted with four foreign doctors working in Ireland, whose feedback strengthened the final questionnaire.

The sampling frame consisted of all 4,965 foreign doctors registered with the Medical Council of Ireland who had a valid email address (96.5 % of those were eligible to participate). A sample size of 357 doctors was sought to provide a ±5 % margin of error for key prevalences, assuming a population of 5,000 eligibledoctors, using the Survey System sample size calculator (http://surveysystem.com/sscalc.htm). In line with previous surveys on migrants in Ireland [15, 16], we anticipated a response rate of approximately 20 %. The Medical Council of Ireland sent an email including a hyperlink to the self-administered questionnaire as well as information to ensure informed consent to an initial random sample of 1,815 potentially eligible doctors, i.e. those whose had registered as non-Irish/EEA nationals. Based on the low response rate, the email was sent to a further 1,194 doctors, making a total of 3,009.

Respondents were asked “in terms of your future plans, do you intend to ‘remain in Ireland permanently’ or ‘remain in Ireland temporarily’”. If they selected ‘temporarily’, they were asked what country they intended to migrate to. If the preferred country corresponded with their country of birth, it was recoded as ‘return home’. Country of birth and country of training were highly correlated. The former was chosen for the category ‘return home’, taking into account that the sample included non-EEA nationals who had graduated from Irish medical schools. Hence, the values of the main outcome variable were ‘remain in Ireland’, ‘return home’ or ‘migrate onwards’. The demographic factors included in the statistical analysis were age, sex, country of origin and country of medical school training, aspirations and intentions prior to arrival in Ireland, and experiences during their time working and living in Ireland.

SAS Version 9.2 was used to undertake data management, descriptive statistics, χ^2^ tests, logistic regression, and stepwise discriminant analysis (Wilk’s lambda). Multiple correspondence analyses were used to explore dependence between future intentions and categorical predictor variables. Multiple binary variables were used for multi-category independent variables. A 5 % two-tailed level of significance was used in all statistical tests and modelling.

## Results

### Respondent demographics

There were 483 responses, of which 366 were complete; 117 respondents began but did not complete the on-line survey and, consequently, were not included in the analysis. Hence, the overall response rate of 16 % fell to an effective response rate of 12 %. Overall, 70 % of the respondents were male (Table 1) and 52 % were aged 31–45 years and could be considered to be at an early to mid-career stage; 13 % were aged over 55 years. Doctors from Pakistan constituted the highest proportion of respondents (24 %), whilst 51 % of the foreign (non-EEA) national doctors who had trained in Ireland (16 % of the total sample) were from Malaysia. As noted earlier, Ireland is a destination country for foreign undergraduate medical students, predominantly from Malaysia, the Middle East, Canada and the United States. Respondents were also from South Africa (13 %), Nigeria (11 %) and Sudan (8 %), countries from where the number of doctors registered with the Medical Council of Ireland increased by almost 30-, 10- and 6-fold, respectively, between 2000 and 2010 [5].

**Table 1:** Doctor demographics and future intentions

Demographic variables			Number	Percent
Sex	Male		254	70 %
	Female		108	30 %
Age, years	<30		33	9 %
	31–35		61	17 %
	36–40		58	16 %
	41–45		68	19 %
	46–50		46	13 %
	51–55		42	12 %
	>56		45	13 %
Country of training	Pakistan		87	24 %
	Ireland	Total	59	16 %
		Born in Malaysia	30	51 %
		Born elsewhere	29	49 %
	South Africa		46	13 %
	Nigeria		40	11 %
	Sudan		29	8 %
	Other high-income countries		23	6 %
	Other low- and middle-income countries		80	22 %
Years spent in Ireland	<2		38	12 %
	3–5		46	15 %
	6–10		97	32 %
	11–15		62	20 %
	>15		63	21 %
Future intentions	Remain in Ireland		105	30 %
	Return home		79	23 %
	Migrate onwards		161	47 %
	Destination	United Kingdom	41	25 %
		United States	26	16 %
		Canada	25	16 %
		Australia	21	13 %
		India	15	9 %
		South Africa	14	9 %
		Other country	15	9 %
		Undecided	4	2 %
Total			366^a^	100 %

^a^Missing data = 2 (country of training), 4 (sex), 13 (age), 21 (future intentions), and 60 (lack of data regarding years spent in Ireland mainly among the 46 South African doctors who appeared to be spending short periods of time in ‘locum’ jobs in Ireland)

### Reasons for migration to Ireland, prior years of experience, years in Ireland and migration intentions

Among the 345 doctors who responded to the question on future plans, 30 % intended to remain in Ireland, whilst 23 % intended to return home and 47 % intended to migrate onwards. The top four destination countries were all English-speaking, including the United Kingdom (25 %), the United States (16 %), Canada (16 %) and Australia (13 %). Together, they accounted for 70 % of those planning to migrate onward to a new country. The four Likert scale response categories on reasons for migration were combined to produce a binary variable: ‘very important’ and ‘important’ as one category versus ‘somewhat important’ and ‘not important’. Table 2 includes the four reasons for migrating to Ireland. There were statistically significant differences in the associations between migration intention, namely to remain in Ireland, return home, or migrate onwards, and the following reasons for migrating to Ireland: ‘to obtain postgraduate qualifications’ (which was highly correlated with the reason ‘for career progression’), ‘for higher salary’ and ‘for family reasons’.

**Table 2:** Reasons for coming to Ireland, prior experience and years spent in Ireland compared with migration intentions

Reasons/Intentions	Migration intentions				
	Intends to remain in Ireland *n* (%)	Intends to return home *n* (%)	Intends to migrate to onwards *n* (%)	Total *n* (%)	*P* value^a^
Reasons for coming to Ireland^b^					
For post-graduate medical qualifications^c^	86 (83 %)	52 (68 %)	122 (81 %)	260/331 (79 %)	0.0236
For higher salary^c^	30 (32 %)	25 (33 %)	79 (52 %)	134/324 (41 %)	0.0018
For family reasons^c^	43 (47 %)	20 (27 %)	48 (32 %)	111/315 (35 %)	0.0161
For safety/security^c^	47 (51 %)	28 (38 %)	69 (47 %)	144/315 (46 %)^d^	0.2502
Years of experience prior to arrival					
<5	8 (8 %)	16 (20 %)	15 (9 %)	39 (11 %)	0.0043
6–10	12 (12 %)	12 (15 %)	37 (23 %)	61 (18 %)	
11–15	20 (19 %)	17 (22 %)	28 (18 %)	65 (19 %)	
16–20	15 (14 %)	4 (5 %)	29 (18 %)	48 (14 %)	
21–25	21 (20 %)	8 (10 %)	18 (11 %)	47 (14 %)	
>25	28 (27 %)	22 (28 %)	33 (21 %)	83 (24 %)	
TOTAL	104 (100 %)	79 (100 %)	160 (100 %)	343 (100 %)^d^	
Years spent in Ireland					
<2	8 (9 %)	12 (20 %)	17 (13 %)	37 (13 %)	0.0054
3–5	5 (5 %)	9 (15 %)	29 (22 %)	43 (15 %)	
6–10	31 (33 %)	16 (27 %)	43 (32 %)	90 (31 %)	
11–15	22 (23 %)	12 (20 %)	26 (19 %)	60 (21 %)	
>15	28 (30 %)	10 (17 %)	19 (14 %)	57 (20 %)	
Total	94 (100 %)	59 (100 %)	134 (100 %)	287 (100 %)^e^	

^a^χ^2^ test of association

^b^Reasons for coming to Ireland are not mutually exclusive

^c^Indication of ‘Very important’ or ‘Important’

^d^Missing data for ‘reasons for coming to Ireland’ and ‘years of prior experience’ range from 0 (345 responses) to 30 (315 responses) respondents

^e^Missing data for years spent in Ireland = 58 – see text

Among the 105 respondents who planned to remain in Ireland, most cited ‘to obtain postgraduate qualifications’ (83 %), ‘safety/security’ (51 %), and ‘family reasons’ (47 %) as reasons for initial migration to Ireland. Among the 79 who intended to return home, a lower proportion reported career and personal/family reasons as very important or important reasons for coming to Ireland compared with those who planned to remain or migrate onwards. Among the 161 respondents who planned to migrate onwards, a similar proportion to those planning to remain in Ireland reported ‘to obtain postgraduate qualifications’ (81 %) as important or very important, while a higher proportion (52 %) cited ‘for higher salary’ as a reason for the initial migration. The variables that were not significantly associated with future plans included sex, marital status, having dependent children, and a wish to live abroad. The largest numbers of non-EEA-trained doctors who planned to remain in Ireland (data not tabulated) were those from Pakistan (*n* = 31, 38 %) and Nigeria (*n* = 17, 45 %). Of the 57 non-EEA respondents (half of whom were from Malaysia) who had qualified as doctors in Ireland and who reported their plans, 42 % intended to migrate onwards to a new destination country, 35 % planned to return home and 18 % planned to remain in Ireland.

The age of respondents (Table 1) and their number of ‘prior years of experience’ before migrating to Ireland were closely correlated; Table 2 uses the latter variable to measure associations with future intentions. Overall, 24 % of the respondents had over 25 years’ prior experience and 47 % of those who planned to remain in Ireland had over 20 years’ prior experience. Of the 79 doctors who planned to return home, 57 % had less than 15 years’ prior experience and 28 % had more than 25 years’ experience before coming to Ireland. Of those intending to migrate to a new destination country, the highest proportion, according to 5-year age categories, was in those with 6–10 years’ prior experience (23 %).

Among respondents who reported their future plans (*n* = 345), 17 % did not respond to the question regarding how much time respondents had spent in Ireland, 69 % of whom were doctors who had trained in South Africa. The association of time in Ireland with future intentions among the 287 doctors who responded to both questions was still statistically significant (*P* = 0.005): 53 % of those who planned to remain in Ireland had been in the country for over 10 years, 47 % of those who planned to return home had been in Ireland for 6–15 years and, perhaps surprisingly, 50 % of those planning to migrate onwards had more than 15 years’ prior experience and 34 % had spent over 10 years living in Ireland.

### Associations of respondents’ experiences in Ireland with migration intentions

Table 3 presents the associations of respondents’ migration intentions with their career progression in Ireland (whether the respondents had applied for and gained entry to a postgraduate training scheme), experiences of training, supervision and career opportunities in Ireland, citizenship status, contract length, and overall satisfaction with life in Ireland. The findings demonstrate some of the factors that may explain migration intentions: 20 % were currently enrolled in and 22 % had completed a postgraduate training scheme. Those who planned to return home were less likely to have enrolled in or completed a training scheme (32 %), while similarly higher proportions of those who planned to remain in Ireland (45 %) and those who planned to migrate onwards (46 %) had been enrolled in or had completed a training scheme. Apart from ‘training scheme’, all associations with migration intentions were statistically significant.

**Table 3:** Experiences in Ireland compared with current intention to stay, return home or migrate onwards

Experiences in Ireland		Migration intentions				
		Intends to remain in Ireland (*n* = 105) *n* (%)	Intends to return home (*n* = 79) *n* (%)	Intends to migrate onwards (*n* = 161) *n* (%)	Total (*n* = 345) *n* (%)	*P* value^a^
Applied for training scheme in Ireland^b^	Yes – Unsuccessfully	25 (24 %)	23 (29 %)	44 (28 %)	92 (27 %)	0.3785
	Yes – Currently on scheme	20 (19 %)	13 (17 %)	36 (23 %)	69 (20 %)	
	Yes – Completed	27 (26 %)	12 (15 %)	37 (23 %)	76 (22 %)	
	No	31 (30 %)	30 (38 %)	42 (26 %)	103 (30 %)	
	Total	103 (100 %)	78 (100 %)	159 (100 %)	340 (100 %)	
I have received adequate training^d^		77 (78 %)	39 (51 %)	91 (60 %)	207/328 (63 %)	0.0006
There are training opportunities for me^d^		56 (58 %)	32 (42 %)	66 (43 %)	154/325 (47 %)	0.0374
I have received adequate supervision^d^		72 (73 %)	44 (57 %)	88 (58 %)	204/327 (62 %)	0.0255
I have opportunities for career progression^d^		47 (48 %)	27 (36 %)	36 (23 %)	110/327 (34 %)	0.0002
Is currently in a contract of 1 year or less^d^		34 (33 %)	34 (44 %)	75 (49 %)	143/334 (42 %)	0.05
Have applied for citizenship immigration procedures^a, c^	Yes	29 (28 %)	14 (18 %)	32 (20 %)	75 (22 %)	<0.0001
	No	21 (20 %)	45 (57 %)	88 (55 %)	154 (45 %)	
	Already hold it	55 (52 %)	20 (25 %)	41 (25 %)	116 (34 %)	
	Total	105 (100 %)	79 (100 %)	161 (100 %)	345 (100 %)	
Overall satisfaction with Ireland^e^		94 (91 %)	57 (73 %)	100 (63 %)	251/340 (74 %)	<0.0001

^a^χ^2^ test of association

^b^Missing data: 5 values

^c^Missing data range from 17 (328 responses) to 20 (325 responses)

^d^Indicated ‘Strongly Agree’ or ‘Agree’

^e^Indicated ‘Very satisfied’ or ‘Satisfied’

Those who intended to remain in Ireland generally reported more positive experiences than those intending to return home or migrate onwards with respect to adequacy of training, adequacy of supervision, and training and career progression opportunities (Table 3). A factor that discriminated between those planning to migrate onwards and those intending to remain in Ireland was a current contract length of 1 year or less. A short current contract was reported by 49 % of those planning to leave, whereas 33 % of those planning to stay held short contracts. Irish citizenship was held by 20 % of potential leavers and by 52 % of those planning to remain. Overall levels of satisfaction with life in Ireland were generally high. They were highest (at 91 %) among those planning to stay in Ireland, at 73 % among those who planned to return home and at 63 % among those who planned to migrate onwards. The five most common specialties currently occupied (data not tabulated) were Medicine (*n* = 61, 19 %), Surgery (*n* = 45, 14 %), General Practice (*n* = 45, 14 %), Anaesthesia (*n* = 35, 11 %) and Paediatrics (*n* = 23, 7 %). A small proportion of respondents (4 %, *n* = 12) were not working at the time they completed the survey.

**Fig. 1:**
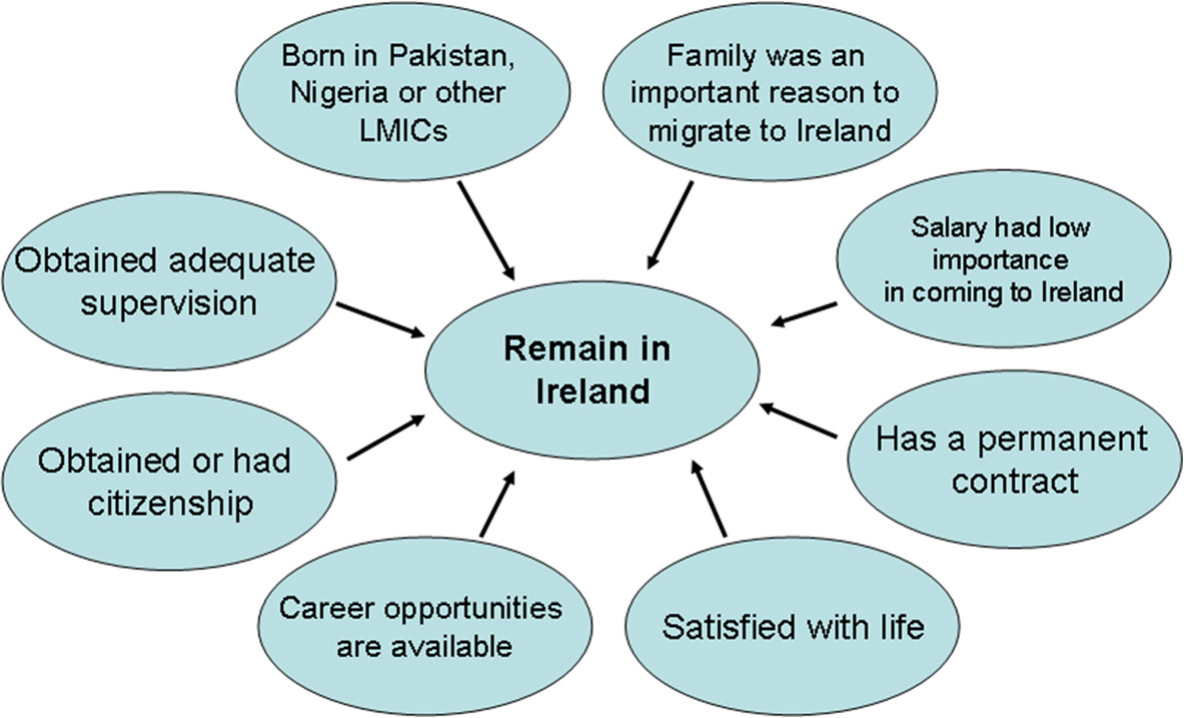
Factors associated with the future intention to remain in Ireland. LMIC, Low- and middle-income country

**Fig. 2:**
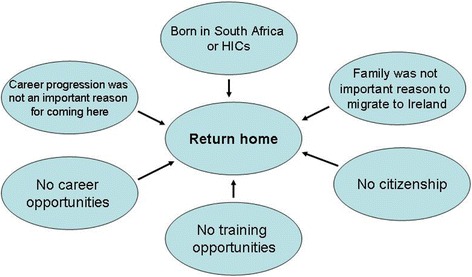
Factors associated with the future intention to return home. HIC, High-income country

**Fig. 3:**
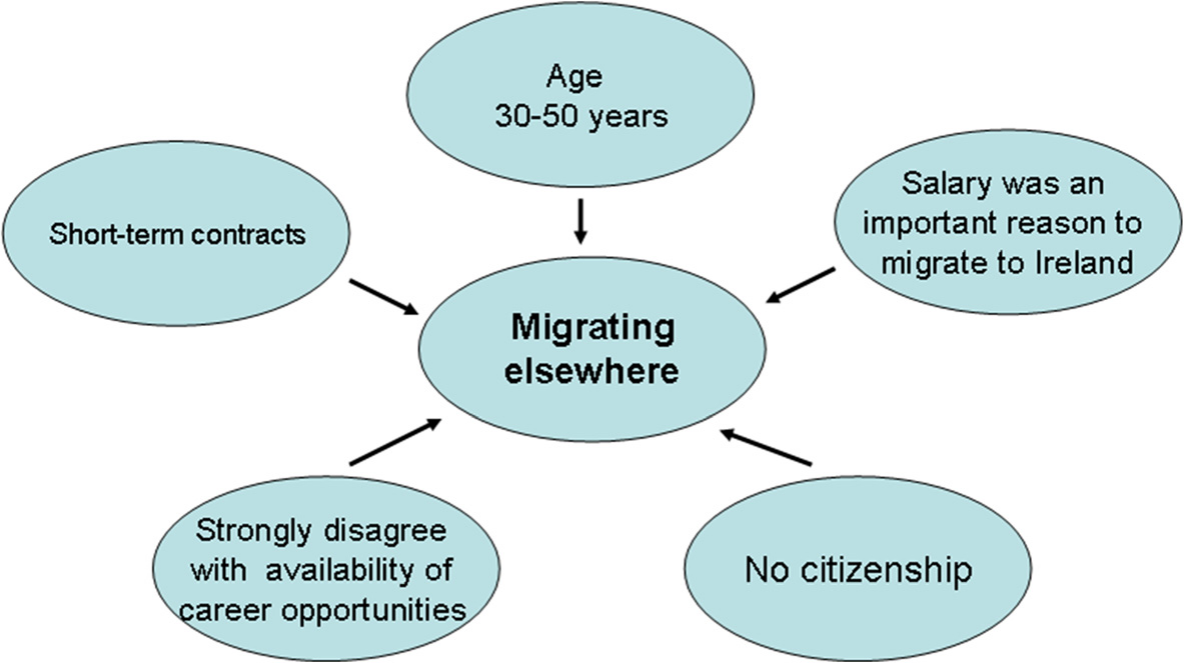
Factors associated with the future intention to migrate onward

Univariate logistic regression analyses identified several demographic variables (age, country of birth, and family, salary and career reasons for migrating to Ireland) to be predictive of future intentions. Variables related to respondents’ experiences in Ireland (availability of career and training opportunities, adequacy of supervision, contract type, salary, satisfaction with life in Ireland, citizenship) were also predictive. Figures 1, 2 and 3, based on discriminant analysis, illustrate the factors associated with migration intentions. Multiple logistic regression analyses (Additional file 1) showed that many factors remained statistically significant when modelled in combination. Stepwise discriminant analyses showed no redundancy of factors (Wilk’s lambda *P* <0.05) with the exception of country of birth. Multiple correspondence analyses of the categorical predictors also showed similar results, with certain categories of predictor variables (e.g. groups of countries of birth) associated with particular future intentions.

Being from a low- or middle-income country (notably Pakistan and Nigeria), family reasons for migrating, positive professional experiences in Ireland (having a permanent contract, career opportunities and adequacy of supervision), citizenship, and satisfaction with life in Ireland were associated with an intention to remain in Ireland (Fig. 1). Being from a high-income country or South Africa, somewhat negative professional experiences, and lack of citizenship were associated with an intention to return home (Fig. 2). Salary as a reason for migrating, more negative professional experiences, and less satisfaction with life in Ireland stood out in the case of those intending to migrate to a new destination country (Fig. 3). Two factors discriminated across all three decision options: citizenship (held by twice as many of those planning to remain) and respondents’ experiences regarding the availability of career opportunities in Ireland. Those staying agreed that such opportunities were available to them in Ireland, whereas those returning home and those migrating onwards disagreed and strongly disagreed with this statement, respectively. The experience of career opportunities, rather than evidence of career progression – through starting or completing a postgraduate training programme – distinguished those who planned to migrate onwards from those intending to remain in Ireland.

## Discussion

The findings herein support earlier propositions that onward migration from the initial destination country may be common [1, 3], in that almost half of survey respondents in this study intended to migrate onwards. The study demonstrates a diverse spectrum of doctors who migrated for different combinations of personal and professional reasons, whose longer-term plans reflect their countries of origin, reasons for migrating, and their positive or negative experiences and perceptions of career opportunities in the destination country. Similar proportions of those planning to migrate onwards (46 %) and those planning to remain in Ireland (45 %) had started or completed postgraduate qualifications. However, most of those who planned to migrate onwards – over a third of whom had been in Ireland for over 10 years – reported that a lack of career progression opportunities for them in Ireland was the main reason for leaving.

An intention to migrate onwards (Fig. 3) may have arisen due to career obstacles experienced after initial formal postgraduate training, and/or because those doctors who had completed a training scheme had achieved their goal and were consequently planning to migrate onwards to new pastures. The beneficiary country was likely to be another high-income English-speaking country, as preferred by 70 % of foreign doctors who planned to migrate on from Ireland. These doctors could be considered to comprise variants of ‘career oriented migrant’ and ‘backpacker’ type migrants [17, 18]. The findings in this paper show that onward migration, rather than returning home after having acquired training in another country, as has been previously proposed [19], may be the norm for some career-oriented migrants. Those who planned to remain in Ireland (Fig. 1) differed not only in their professional experiences but also in their migration hopes and aspirations. Unlike those who planned to migrate onwards, salary was not an important reason for migrating, whereas family reasons were important. Doctors who planned to return home (Fig. 2) had generally not migrated to progress their careers. Nevertheless, they reported a lack of training and career opportunities in Ireland, illustrating how perceived shortcomings in the medical workforce environment in the destination country contribute to the loss of doctors. It is likely that some, such as those originating from South Africa, may have spent short periods of time (weeks or months) in Ireland on ‘locum’ jobs or covering for staff on leave, corresponding to the ‘commuter’ migrant type [17].

Over two thirds of those planning to return home had not obtained a place on a postgraduate training scheme, which suggests that the source country was less likely to benefit from their return home. Given its high reliance on the international recruitment of foreign doctors, this finding questions Ireland’s implementation of Article 3.8 of the WHO Global Code on the International Recruitment of Health Personnel, which states that, “*Member states should facilitate circular migration of health personnel, so that skills and knowledge can be achieved to the benefit of both source and destination countries*” [20]. Among the 25 recommendations suggested in a recent Strategic Review of Medical Training and Career Structure from Ireland’s Department of Health [21, 22] is one that specifically addresses the issue of the 900 service posts that are mainly occupied by foreign doctors. Service post-holders stay indefinitely, on short contracts, often moving annually (with their families) between small rural hospitals [7], where there are few specialists to ensure supervision and the maintenance of standards. However, one study finding that might surprise Irish national stakeholders is that the provision of formal postgraduate training might not be sufficient to retain foreign doctors if the broader systemic factors that cause dissatisfaction among Irish and foreign-trained doctors working in Ireland are not tackled [21, 22]. Twice as many of the foreign doctors who had started or completed postgraduate training planned to migrate onwards compared with the proportion among those planning to return home.

A similar experience – dissatisfaction with career progression opportunities – has been reported by Irish-trained health professionals who emigrated from Ireland between 2008 and 2013 [14, 23] (in this Supplement). Together, the findings from these two studies point to a huge challenge for the Irish health system. Ireland has increased the inflow of doctors by doubling the intake of Irish entrants into medical schools between 2007 and 2012 and by increasing the recruitment of foreign-trained doctors. However, the increased outflow appears to be matching the increased inflow. High levels of onward migration by foreign-trained doctors and emigration by locally-trained doctors are a barometer of the effectiveness of health workforce retention policies in destination countries. The reasons cited by Irish doctors who had emigrated [14] (dissatisfaction with career opportunities, career progression, training and salary) and the reasons cited by the foreign doctors in this study who planned to migrate onwards (lack of career opportunities, short-term contracts and salaries as a reason for migrating to Ireland), suggest that these two sets of doctors have more in common than what distinguishes them. International recruitment is not a desired policy given Ireland’s commitment to the WHO Global Code [20] and the doubling of medical training capacity in Ireland; nonetheless, many foreign doctors have spent several years or decades working in Ireland. It is likely that some of the measures needed to retain Irish doctors [21, 22] would also reduce the onward migration of foreign doctors to other English-speaking countries, many of whom had spent over 10 years in Ireland.

Since 2013, Ireland has taken new steps to implement Article 3.8 of the WHO Global Code through an innovative bilateral relationship (Code Article 5.2) between Ireland’s and Pakistan’s national training colleges [24]. The International Medical Graduate Training Initiative is providing a 2-year postgraduate training programme in Irish hospitals to three intakes (*n* = 90) of doctors from Pakistan, selected by Pakistan’s College of Physicians and Surgeons, covering specialties where there is a need for postgraduate training in Pakistan. On successful completion, the doctors must return to Pakistan to complete their specialist training and be awarded their postgraduate training certificates. The advantage for Ireland is that the initiative has encouraged national training bodies to ensure that accredited training and close supervision is delivered across a wider range of Irish hospitals, thereby resulting in an improved hospital service to Irish rural populations. Its effectiveness in achieving the goal of training Pakistani doctors to return and remain in Pakistan will be the subject of a forthcoming evaluation.

### Study limitations

The chief limitation of the paper is the low response rate of 16 %, with an effective response rate of completed questionnaires of 12 %, which means that the findings may not be representative of all foreign doctors in Ireland. Despite this, the sample compares well with the profile of non-Irish/EEA doctors registered with the Medical Council of Ireland, apart from over-representation of those aged 31–35 years (17 % vs. 14 %) and under-representation of those aged 46–50 years (13 % vs. 16 %). Percentages by country of qualification were similar, the main difference being an under-representation of Sudan (8 % vs. 11 %) and South Africa (13 % vs. 15 %) and over-representation of Nigeria (11 % vs. 8 %). Analysis of 2013 Medical Council registration data, cross-tabulated by ‘where did you practice medicine in the last 12 months?’, provides some explanation for the low response rate: only 2186 (59 %) of the 3,685 registered non-EEA graduates had practiced solely within Ireland during this period and 27 % had practiced outside of Ireland in the previous year. Hence, many registrants might not have received the survey or, alternatively, would not have seen it as relevant to their current circumstances. Secondly, the validity of the findings is supported by our earlier qualitative paper [7], which reported similar dissatisfaction with training and career opportunities in Ireland, especially among respondents who reported plans to migrate to a new destination country. Additionally, the reasons why many doctors do not wish to fulfil their careers in Ireland are plausible and consistent across studies [7, 18, 23].

A limitation of the cross-sectional study design involving a survey of foreign doctors, up to 40 % of whom had been in Ireland for over 10 years, is that recall bias is likely given that respondents were reporting reasons for a migration decision made, in many cases, over a decade previously. The direction of the bias may have resulted in doctors ascribing, as reasons for the original migration, those that were now important to them such as a desire to access postgraduate training given its importance in obtaining a permanent post. Finally, there are limits with respect to the interpretation of expressed intentions of foreign doctors to migrate onwards or return home [25, 26]. Responses to attitudinal questions around training, career and overall satisfaction provide explanatory variables that may predict intended behaviour. However, in the first instance they are indicators of foreign doctors’ levels of satisfaction or dissatisfaction with the types of posts in which they are working, and associated difficulties of settling and building a stable life in the destination country. Self-reported intentions to leave can be considered as the first of a series of steps. Subsequent steps, showing that intentionality is being acted upon, include requests for verification of qualifications by professional council registries in destination countries [19]. The final steps – evidence of registration and employment in the destination country – provide confirmation. However, the importance of these findings for health workforce planners in Ireland lies in how foreign doctors, similarly to Irish doctors, perceive the desirability of careers in the Irish health system.

## Conclusion

This paper reports diverse patterns in foreign doctors’ backgrounds and reasons for migrating to Ireland, their experiences in Ireland, and their future intentions. These cluster around the three options open to them, namely migrate onwards, remain in the current destination country, or return home. Close to half of the participants intended to migrate to a new destination country, often after a decade or more of living and working in Ireland, for similar reasons to those why Ireland is struggling to retain the doctors it trains [14]. Large scale emigration of host country-trained doctors and onward migration by internationally recruited doctors have the same root causes – systemic weaknesses in how the country treats its medical workforce. In the case of Irish doctors who had left and foreign doctors who were planning to leave, dissatisfaction with training and career opportunities was the root cause, aggravated by the consequences of the economic downturn for doctors’ salaries. Measures to address these root causes have been agreed [21, 22]. If implemented, these are likely to help retain both locally- and foreign-trained doctors. This will thereby reduce the need for future international recruitment, improving the self-sufficiency of Ireland’s medical workforce. Innovative models for providing postgraduate training to foreign-trained doctors, tailored to meet the needs of the source countries, can further enable destination countries to implement the WHO Global Code on the International Recruitment of Health Personnel.

## Additional file

Additional file 1: Results of logistic regression analyses. **Table S1.** Factors associated with intention to remain in Ireland. **Table S2.** Factors associated with intention to return home. **Table S3.** Factors associated with intention to migrating onwards/elsewhere. (DOC 115 kb)
