# Supplementary material for: Passing through – reasons why migrant doctors in Ireland plan to stay, return home or migrate onwards to new destination countries
Source: Hum Resour Health. 2016 Jun 30;14(Suppl 1):35. doi: 10.1186/s12960-016-0121-z (PMC4943478; doi:10.1186/s12960-016-0121-z)
Supplement: Additional file 1: — Results of logistic regression analyses. Table S1. Factors associated with intention to remain in Ireland. Table S2. Factors associated with intention to return home. Table S3. Factors associated with intention to migrating onwards/elsewhere. (DOC 115 kb) [file 12960_2016_121_MOESM1_ESM.doc]

**Results of Logistic Regression Analyses**

**Additional file 1: Table S1. Factors associated with intention to remain in Ireland**

| Factor | Univariate analysis | | Multiple regression analysis | |
| --- | --- | --- | --- | --- |
|  | OR ^a^ | *P* value | Adjusted OR ^a^ | *P* value |
| Born in Pakistan, Nigeria or other LMIC | 1.98 | 0.049 | 1.29 | 0.630 |
| Family was an important reason to migrate to Ireland | 2.00 | 0.008 | 3.70 | <0.001 |
| Salary had low importance in migrating to Ireland | 1.80 | 0.022 | 1.39 | 0.373 |
| Has a permanent contract | 3.00 | 0.002 | 1.88 | 0.180 |
| Obtained adequate supervision | 2.04 | 0.007 | 1.33 | 0.438 |
| Obtained or had citizenship | 4.97 | <0.001 | 5.00 | <0.001 |
| Career opportunities are available | 2.49 | <0.001 | 3.00 | 0.003 |
| Satisfied with life | 5.32 | <0.001 | 5.73 | <0.001 |

^a^ Risk of remaining in Ireland relative to returning home or migrating onwards

OR, Odds ratio; LMIC, Low- and middle-income country

**Additional file 1: Table S2. Factors associated with intention to return home**

| Factor | Univariate analysis | | Multiple regression analysis | |
| --- | --- | --- | --- | --- |
|  | OR ^a^ | *P* value | Adjusted  OR ^a^ | *P* value |
| Born in South Africa or HICs | 3.33 | 0.002 | 1.37 | 0.599 |
| Family was not an important reason to migrate to Ireland | 2.41 | 0.008 | 5.56 | <0.001 |
| Career progression was not an important reason for migration to Ireland | 2.54 | 0.017 | 5.70 | 0.006 |
| No citizenship | 5.29 | <0.001 | 6.51 | <0.001 |
| No training opportunities | 3.41 | <0.001 | 3.31 | 0.008 |
| No career opportunities | 1.71 | 0.040 | 2.50 | 0.036 |

^a^ Risk of returning home relative to remaining in Ireland

OR, Odds ratio; HICs, High-income countries

**Additional file 1: Table S3. Factors associated with intention to migrating onwards/elsewhere**

| Factor | Univariate analysis | | Multiple regression analysis | |
| --- | --- | --- | --- | --- |
|  | OR ^a^ | *P* value | Adjusted  OR ^a^ | *P* value |
| Aged 30–50 years | 1.68 | 0.024 | 1.21 | 0.632 |
| Salary was an important reason to migrate to Ireland | 2.31 | 0.002 | 1.64 | 0.177 |
| Dissatisfied with life in Ireland | 6.61 | <0.001 | 5.98 | <0.001 |
| Short-term contracts | 1.90 | 0.023 | 1.44 | 0.312 |
| No citizenship | 4.82 | <0.001 | 4.45 | <0.001 |
| Strongly disagree with availability of career opportunities | 3.85 | <0.001 | 2.96 | 0.008 |

^a^ Risk of migrating elsewhere relative to remaining in Ireland
